# Student Perspectives on Professionalism: Time to Reform Curriculum for Better Patient Experience

**DOI:** 10.1177/23743735251401814

**Published:** 2025-12-22

**Authors:** Nagina Khan, Sucheta Tiwari, Walther van Mook, Subodh Dave, Sohyun Ha, Joshua Sagisi, Marie Hickman, Nicole Davi, Chantel Aftab, Salim Al-Huseini, Wolfgang Gilliar

**Affiliations:** 1 Centre for Health Services Studies (CHSS), School of Social Sciences, 2240 University of Kent, Canterbury, Kent, UK; 2 NIHR Applied Research Collaboration Kent, Surrey and Sussex (ARC KSS), UK; 3 4955 Department of Psychiatry, East London NHS Foundation Trust, London, UK; 4 Department of Intensive Care Medicine, 199236 Maastricht University Medical Centre, Maastricht, Netherlands; 5 Faculty of Health, Medicine, and Life Sciences, School of Health Professions Education, Maastricht University, Maastricht, Netherlands; 6 Department of Psychiatry, 2940 Derbyshire Healthcare NHS Foundation Trust, Derbyshire, UK; 7 Department of Psychiatry, Royal College of Psychiatrists, London, UK; 8 College of Osteopathic Medicine, 219177 Touro University Nevada, Henderson, NV, USA; 9 2940 Library Services, Derbyshire Healthcare NHS Foundation Trust, Derbyshire, UK; 10 Department of Psychiatry, 1730 Birmingham and Solihull Mental Health Trust (UK), Birmingham, UK; 11 Department of Psychiatry, Al Masarra Hospital, Ministry of Health, Muscat, Oman; 12 Naples College of Osteopathic Medicine, Tampa, FL, USA

**Keywords:** mixed-methods review, professionalism, undergraduate medical education, patient experience, student perspectives, curriculum development

## Abstract

Professionalism is central to patient experience, however undergraduate medical education often emphasizes standardized frameworks over relational, context-sensitive learning. Students’ perspectives remain underrepresented in curriculum design despite shaping future care delivery. To synthesize undergraduate medical students’ views on professionalism education, identify barriers and enablers to learning, and highlight implications for curriculum reform and patient-centered care. A mixed-methods systematic review was conducted, including qualitative, quantitative, and mixed-methods studies published between 2010 and 2023. Four databases (PubMed, Embase, PsycINFO, Education Resources Information Center [ERIC]) were searched. Quality appraisal used the Mixed-Methods Appraisal Tool and thematic analysis was applied to integrate findings, emphasizing learner voices and links to patient experience. Fifty-four studies were included (38 qualitative, 16 quantitative/mixed methods). Students consistently emphasized the influence of role modeling by senior clinicians, reflective practice, patient narratives, and supportive learning environments. Barriers included inconsistent teaching, cultural dissonance between formal curricula and observed clinical behaviors, and lack of structured feedback. Learning was most impactful when professionalism teaching was embedded in real patient care, reinforced through observation, supervision, and reflection. Students perceive professionalism as relational practices, empathy, respect, integrity, and accountability that directly shape patient experience. Curricula should integrate structured role modeling, reflective exercises, patient narratives, and culturally responsive teaching. Centering student perspectives in curriculum design can better prepare graduates for context-sensitive, patient-centered practice.

## Introduction

Professionalism in healthcare is closely tied to patient experience, shaping how care is delivered, perceived, and trusted. Patients often identify professionalism not in abstract terms, but through relational behaviors: listening, empathy, honesty, respect, and accountability. While these values are formally embedded in medical education curricula, they are often taught through top-down models that prioritize standardization over contextual relevance. Recent scholarship^1,2^ highlights a persistent gap between the intentions of professionalism curricula and the lived experience of students and patients in clinical settings.

Accreditation bodies typically define outcomes and frameworks for professionalism teaching, aiming for national consistency. Yet implementation occurs in diverse environments where institutional culture, resources, interprofessional dynamics, and patient populations vary widely. These contextual factors influence how professionalism is observed, modeled, and enacted ultimately affecting the patient experience.^3,4^ Reform efforts that neglect this complexity risk reinforcing sameness rather than promoting meaningful change.

There is growing recognition that the patient's experience is not shaped solely by technical care, but by the professionalism with which that care is delivered. Recent literature shows that when students are exposed to high-quality role models and supported by inclusive, reflective learning environments, their understanding of professionalism deepens, and their care becomes more person-centered.^5,6^ However, learners frequently encounter dissonance between what is taught and what is practiced. Without adequate space to reflect, question, and codevelop these insights, the learning of professionalism becomes passive and disconnected from its most critical outcome: improving patient experience.

Moreover, student perspectives remain underrepresented in curriculum design, despite their central role in shaping the future health workforce. As coproducers of education, students can offer insight into both the strengths and shortcomings of professionalism teaching. Their lived experiences of learning in clinical spaces positive or otherwise can provide valuable guidance for improving how professionalism is taught and assessed. Coproducing professionalism education with learners is not only an inclusive pedagogical strategy but a patient-centered one: students’ understanding of professionalism often mirrors the care they will one day provide.

This study aims to re-center learner voices in professionalism education and to examine how such perspectives can improve both curriculum design and patient experience. Specifically, we sought to:

- Identify learners’ views on barriers and enablers to learn professionalism in undergraduate education.
- Explore which tutoring methods students perceive as most impactful in developing professional behaviors.
- Inform future curriculum priorities, especially as they relate to preparing students for relational, context-aware practice in a changing healthcare landscape.

In synthesizing qualitative and quantitative studies, we aimed to generate recommendations that support a more integrated, student-informed approach to professionalism education. By embedding the learner voice in curriculum design, we contribute to the broader goal of aligning medical education with the principles of empathy, responsiveness, and respect that define an excellent patient experience.

## Study Design

We conducted a mixed-methods systematic review to explore undergraduate medical students’ perspectives on professionalism education and its impact on patient experience. This approach allowed integration of qualitative, quantitative, and mixed-methods studies, reflecting the complexity of professionalism as a relational and context-dependent construct. Our review adheres to Preferred Reporting Items for Systematic Reviews and Meta-Analyses (PRISMA) 2020 guidelines for systematic reviews^7^ and is registered with the International Registered Report Identifier (IRRID): PRR1-10.2196/37473.

### Search Strategy

The population of interest included undergraduate medical students, as well as medical educators and clinical supervisors involved in teaching and assessing professionalism. We searched PubMed (Medline), Embase, PsycINFO, and Education Resources Information Center (ERIC) for English-language peer-reviewed articles published between January 2010 and December 2023. Search terms combined keywords and MeSH terms related to “professionalism,” “medical students,” “patient experience,” and “undergraduate medical education.” References lists of the included studies were hand-searched to ensure comprehensiveness (see Supplemental Table 1).

### Eligibility Criteria

We included empirical studies employing qualitative, quantitative, or mixed-methods designs that explored undergraduate medical students’ conceptualizations, experiences, or perceptions of professionalism education. Studies were included if they addressed how professionalism was taught, assessed, or understood within undergraduate curricula. Exclusion criteria included: postgraduate/residency-focused studies, nonempirical articles (reviews, commentaries), and studies not directly related to professionalism education.

### Study Selection and Data Extraction

Titles and abstracts were screened using Rayyan, an artificial intelligence-assisted systematic review tool.^8^ Two independent reviewers conducted full-text screening, with disagreements resolved through discussion or third-party adjudication. Data extraction captured study characteristics (author, year, country), objectives, methodology, participant details, and key findings and was performed independently by two researchers using a standardized Excel template.

### Quality Appraisal

The methodological quality of the included studies was evaluated using the Mixed-Methods Appraisal Tool (MMAT),^9^ appropriate for appraising qualitative, quantitative, and mixed-methods research. Two independent reviewers performed quality assessments; discrepancies were resolved via discussion or a third reviewer. Quality appraisal informed the interpretation of findings but did not exclude studies, allowing a comprehensive synthesis of diverse learner perspectives.

### Data Synthesis

Data were analyzed using thematic analysis. Qualitative and quantitative findings were integrated to identify patterns in learners’ understanding and experiences of professionalism, as well as educational strategies linked to patient-centered outcomes. Themes emphasized learners’ voices, contextual factors, and educational methods that most effectively support professional development.

### Ethics Statement

Ethical approval and informed consent were not required, as this study synthesized previously published literature.

**Figure 1.**
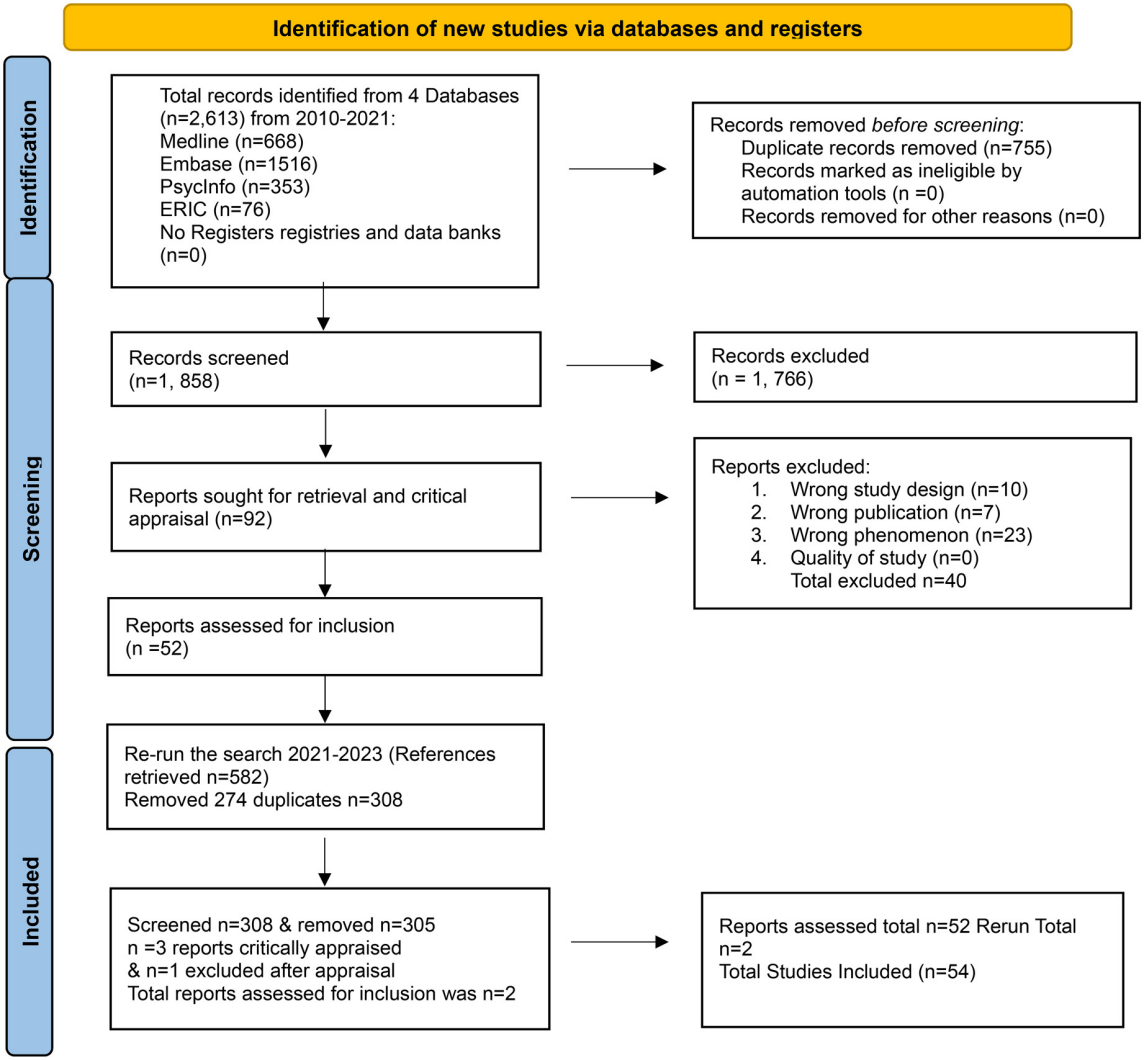
PRISMA 2020 flow diagram (Transparent Reporting of Systematic Reviews and Meta-Analyses, 2015).

Figure 1 displays the PRISMA 2020 flow diagram documenting the search and screening process.^10^

## Results

### Study Selection and Characteristics

The database search yielded 2613 records. After removal of 755 duplicates, 1858 unique titles and abstracts were screened, with 1652 excluded. Ninety-two full-text articles were assessed, of which 40 were excluded due to study design (*n* = 11), publication type (*n* = 7), or irrelevance (*n* = 23). An updated search (2021-2023) identified 382 additional records, 74 of which were excluded. In total, 54 peer-reviewed studies met inclusion criteria and were critically appraised (see PRISMA flow diagram, Figure 1).^7^

A clear temporal trend was observed, with a gradual increase in publications between 2010 and 2023, peaking in 2019. Of the included studies, 38 used qualitative methods and 16 employed quantitative or mixed-methods designs. Key characteristics (authorship, aims, context, design, publication year) are provided in Supplemental Table 2.

### Quality Appraisal Summary

All 54 studies were appraised using the MMAT.^9^ Two reviewers independently assessed each study, resolving disagreements through discussion with a third reviewer (interrater agreement: 96%).

*Quantitative and mixed-methods studies (n = 16)*: Ten met all MMAT criteria, demonstrating clear sampling, valid instruments, and appropriate analyses. Six reported limitations such as low response rates (<60%) or incomplete handling of missing data.
*Qualitative studies (n = 38)*: Thirty studies demonstrated strong methodological rigor, including reflexivity, data saturation, and audit trails. Eight studies lacked discussion of researcher positionality or participant validation.

No studies were excluded based on quality. Identified weaknesses were considered during interpretation.

### Geographical Distribution

Most studies originated from the United States, with additional contributions from Europe, Asia, and the Middle East. Table 1 summarizes the geographical distribution of the included studies.

**Table 1.** Distribution of Studies by Country.

Country/Region	Number of Studies
UAE (Shamim et al, 2016)	1
Saudi Arabia (Shamim et al, 2016)	1
Kuwait (Al-Abdulrazzaq et al, 2014)	1
Iran (Azmand et al, 2018; Mirmoghtadaie et al, 2020; Safari et al, 2020)	3
Türkiye (Kavas et al, 2015)	1
United States (Abrams et al, 2021; Braun et al, 2013; Butani et al, 2019; Curry et al, 2011; Gonsalves and Zaidi, 2016; Hultman et al, 2012; Karnieli-Miller et al, 2010; Karnieli-Miller et al, 2011; Kaul et al, 2014; Maitra et al, 2021; Pierce et al, 2020; Prunuske et al, 2019; Shield et al, 2015; Shield et al, 2011; Tucker et al, 2016; Yoon et al, 2017)	18
United Kingdom and Ireland (Borgstrom et al, 2010; Finn et al, 2010; Kong and Knight, 2017; Stockley and Forbes, 2014; Varga-Atkins et al, 2010)	7
Ireland (Bennett et al, 2013; McEvoy et al, 2012)	
Canada (Cusimano et al, 2019; Ginsburg and Lingard, 2011; Kittmer et al, 2013; Ramakrishna et al, 2014; Shevell et al, 2015; Wang et al, 2019)	6
Australia (Barr et al, 2014; Langendyk et al, 2016; Monrouxe et al, 2011)	3
Germany (Lutz Gabriele, Scheffer Christian, Edelhaeuser Friedrich, Tauschel Diethard, 2013; Shiozawa et al, 2020)	2
Sweden (Fredholm et al, 2019; Haffling et al, 2010)	2
Netherlands (Adema et al, 2019; Mak-van der Vossen et al, 2018)	2
India (Dhaliwal et al, 2018)	1
Collaborative studies (multiple countries) Canada, New York City, United States (Arntfield et al, 2013)	6
Portugal, Brazil, and Netherlands (Ribeiro et al, 2021)	
Taiwan, United Kingdom, and Australia (Shaw et al, 2018)	
Aberdeen, United Kingdom, Netherlands (Stubbing et al, 2019)	
Canada and Norway (Whelan et al, 2021)	
West Indies, Trinidad, and Tobago (Youssef et al, 2016)	
Total	54

### Synthesis of Findings

Thematic analysis by two researchers and four medical students grouped findings into three major themes:

1. Evolving Definitions of Professionalism
  Learners consistently described professionalism as relational and patient-centered, grounded in values such as empathy, honesty, respect, accountability, and integrity. While broad consensus existed across most domains,^11,12^ advocacy and commitment to education were less frequently emphasized.^13^ Empathy was identified as the most essential competency.^14^ Students noted, however, that professionalism was often underemphasized in formal assessments despite its importance. Concepts such as trust, encouragement, and role modeling were described as the “soul” of professionalism.^15^
2. Learning through Role Modeling, Observation, and Supervision
  Authentic clinical experiences and supervised patient interactions were central to how students internalized professional values.^16,17^ Senior clinicians’ behaviors both positive and negative were identified as powerful influences, often outweighing formal teaching.^18–20^ Negative role modeling and hidden curricula sometimes undermined learning, contributing to distress, shame, or burnout.^21^ Students emphasized the need for structured observation, constructive feedback, and opportunities for reflective discussion to support professional growth.^22^
3. Effective Educational Strategies for Professionalism Development
  Students valued learning approaches that connected professionalism with real patient care. Narrative medicine methods (eg, reflective journaling, essays, storytelling) were frequently reported as effective for developing empathy, communication skills, and identity formation.^23,24^ Small-group discussions of clinical incidents encouraged critical reflection.^25^ Peer learning and patient narratives reinforced the humanistic dimensions of care. Role modeling by faculty, patients, and peers reinforced positive behaviors, while reliance on instincts alone was seen as insufficient^26^ (Table 2).

Table 3 shows what works, how to embed it in curricula, and how to measure it: the good practices and actionability.

**Table 2.** Learner-Identified Professionalism Constructs and Preferred Teaching Methods.

(a) Learner-Identified Constructs	(b) Learner Activities Linked to Constructs	(c) Learner Preferred Teaching Methods
Professional behaviors: patience, honesty (Hultman et al, 2012) Respect (Karnieli-Miller et al, 2010, 2011) Patient care—altruism (Karnieli-Miller et al, 2011) Communication (Shield et al, 2015) Accountability, duty, excellence, honor, integrity (Hultman et al, 2012) Authenticity (Shevell et al, 2015) Collaboration (Abdalla et al, 2020; Kavas et al, 2015; Ramakrishna et al, 2014; Shield et al, 2011) Commitment to patients, profession, and society (Al-Abdulrazzaq et al, 2014) Empathy as most important competency (Gonsalves and Zaidi, 2016) Trust in team interactions and relationship satisfaction (Azmand et al, 2018; Stubbing et al, 2019) Attachment to patients, supervisors, and workplace (Fredholm et al, 2019) Principle-based attitudes and emotional intelligence (Butani et al, 2019) Humanism toward others and self (Butani et al, 2019) Stressful environments, poor interprofessional collaboration, and emotional exhaustion (Mirmoghtadaie et al, 2020; Wang et al, 2019) Shame experiences (Whelan et al, 2021) Professional identity formation and humanistic qualities (Abrams et al, 2021; Finn et al, 2010; Shaw et al, 2018) Patient-centered agenda and managing student–patient conflict (Barr et al, 2014; Borgstrom et al, 2010) Ethical practice and moral dilemmas (Ribeiro et al, 2021; Haffling et al, 2010; Langendyk et al, 2016) Role modeling, leniency, and sacrifice (Finn et al, 2010)	Enhance capacity to collaborate, empathize, be patient-centered, and personal development (Arntfield et al, 2013) Developing communication skills (Arntfield et al, 2013) Navigating emotional patients and trainee role (Maitra et al, 2021) Meaningful workplace social interactions (Adema et al, 2019; Song and Elftman, 2023) Managing workload and well-being (Azmand et al, 2018; Borgstrom et al, 2010) Managing moral and ethical dilemmas (Ribeiro et al, 2021) Engaging in professional identity formation (Finn et al, 2010)	Narrative medicine (Arntfield et al, 2013; Dhaliwal et al, 2018; Pierce et al, 2020) Reflective writing and essays (Abrams et al, 2021; Arntfield et al, 2013; Barr et al, 2014; Bennett et al, 2013; Braun et al, 2013; Butani et al, 2019; Kittmer et al, 2013; Langendyk et al, 2016; Shaw et al, 2018; Shield et al, 2011; Shiozawa et al, 2020; Varga-Atkins et al, 2010; Yoon et al, 2017) Small-group discussions on critical incident reports (Kittmer et al, 2013; Kong and Knight, 2017; Varga-Atkins et al, 2010) Case-based sessions (Shield et al, 2015) Hidden curriculum exploration (Azmand et al, 2018; Wang et al, 2019) Near-peer facilitation (Cusimano et al, 2019; Prunuske et al, 2019) Role modeling (Curry et al, 2011; Finn et al, 2010; Safari et al, 2020) Medical–clinical interaction storytelling (Karnieli-Miller et al, 2011) Use of virtual and emotional patients (Azmand et al, 2018; Maitra et al, 2021; McEvoy et al, 2012) Videotaped scenarios (Ginsburg and Lingard, 2011) Mentoring, lectures, and journal clubs (Hultman et al, 2012) Portfolio-workbook (Shamim et al, 2016) Remediation for lapses in professionalism (Kaul et al, 2014; Mak-van der Vossen et al, 2018; Tucker et al, 2016) Avoiding “ticking-box” approaches and fostering engagement (Stockley and Forbes, 2014) Contact with positive role models, patients, families, and peers (Al-Abdulrazzaq et al, 2014)

**Table 3.** Good Practices in Professionalism Education: Evidence, Curricular Applications, and Measurable Outcomes.

Practice Identified by Students	Description/Evidence From Studies	Suggested Curricular Application	Potential Measurable Outcomes
Narrative medicine and reflection	Reflective journaling, essays, and storytelling enhanced empathy, ethics, and identity formation (Arntfield et al, 2013; Dhaliwal et al, 2018).	Embed structured reflective writing sessions into clinical rotations; use guided prompts.	Increased empathy scores: improved reflective capacity (measured via validated scales).
Role modeling and supervision	Senior clinicians’ behavior strongly influenced student professionalism; both positive and negative models noted.^18,27^	Faculty development for explicit role modeling; structured observation with feedback.	Student-reported quality of role modeling; alignment between observed and taught values.
Patient narratives and early contact	Direct engagement with patients normalized a patient-centered identity and fostered empathy.^28^	Incorporate expert patient teachers and early patient contact into preclinical years.	Patient satisfaction with student interactions; student empathy ratings.
Small-group discussion of critical incidents	Peer and faculty-facilitated discussions encouraged reflection on professionalism challenges.^20,25^	Regularly scheduled professionalism “case rounds” or ethics discussions.	Depth of reflection (Rubric-based); confidence in handling ethical dilemmas.
Teamwork and collaboration exercises	Students linked professionalism to communication and accountability in teams.^29^	Interprofessional simulation sessions with structured debriefs.	Teamwork assessment scores; peer evaluations.
Cultural responsiveness	Professional identity shaped by local culture and societal expectations.^22^	Integrate modules on sociocultural influences in professionalism.	Student cultural humility/self-awareness scores.

## Discussion

This mixed-method review of 54 studies highlights how professionalism in medical education is deeply intertwined with patient experience and the quality of clinician–patient relationships. Importantly, students’ voices reveal not only how professionalism is defined and learned, but also which teaching practices translate into meaningful patient-centered behaviors.

Our findings emphasize that professionalism is experienced relationally through respect, integrity, and communication in everyday clinical encounters. Respect emerged as a core dimension shaping patient experience, expressed in how learners honor patients’ time, preferences, and dignity.^30^ Conversely, lapses such as breaches of confidentiality or unprofessional behaviors were seen as directly undermining patient trust.^31^ Learners’ reflections show that professionalism is not only about ethical knowledge but also about moral courage and supportive supervision that enables students to act in line with values during patient care.^32^

### Role of Role Modeling and Supervision

One of the most consistent findings was the influence of senior clinicians and faculty role modeling. Positive role models demonstrated empathy, accountability, and integrity, reinforcing professionalism through lived example.^18,19^ Conversely, poor role modeling or hidden curricula led to confusion, distress, and disengagement.^27^ Embedding structured observation and feedback into curricula could help make this influence intentional and constructive (see Table 3).

### Cultural and Contextual Influences

Professional identity formation was also shaped by local culture, social values, and institutional norms. Learners described professionalism differently across settings, often linking it to societal expectations of doctors in their specific context.^22^ This underscores the need for curricula that acknowledge cultural diversity and prepare students to adapt professionalism to different patient populations.

### Good Practices for Curriculum Reform

To support actionable reform, we synthesized effective practices across studies (Table 3). Narrative medicine, structured reflection, patient narratives, and interprofessional teamwork consistently fostered professional growth. Small-group critical incident discussions created safe spaces for grappling with ethical dilemmas, while cultural responsiveness training helped learners contextualize professionalism in diverse care settings. Mapping these practices to curricular applications and measurable outcomes provides educators with a practical roadmap for embedding professionalism training.

### Emotional Complexity and Psychological Safety

Students also reported the emotional complexity of professionalism learning, including shame, fear of failure, and distress when values clashed with institutional practices.^33,34^ Importantly, no positive outcomes of shame were identified, reinforcing the importance of psychological safety in clinical education. Creating supportive environments that normalize reflection and error discussion without stigma is crucial to sustaining learner engagement and patient-centered care.

### Strengths and Limitations

A key strength of this study is its mixed-methods approach, integrating quantitative and qualitative perspectives to generate a comprehensive picture. Student involvement in both the design and analysis stages enhanced the practical relevance of our synthesis. Limitations include reliance on published reports without access to raw data (eg, interview transcripts), which may have constrained depth of interpretation. Additionally, this review focused exclusively on undergraduate medical students; postgraduate learners were excluded to maintain scope and comparability across studies. While this allowed us to center undergraduate perspectives, it does limit insights into later stages of professional identity formation. Future reviews should expand to postgraduate contexts.

## Conclusion

This systematic review highlights that undergraduate medical students understand professionalism as relational, grounded in empathy, respect, and accountability toward patients. Their learning is strongly shaped by role modeling, supervised patient interactions, and supportive reflection, while misalignment between formal curricula and observed clinical practice can hinder professional growth.

To strengthen professionalism education, educators should:

1. Embed structured observation and feedback from senior clinicians into clinical placements.
2. Integrate narrative medicine, reflective practice, and patient narratives to link professionalism to patient experience.
3. Incorporate cultural and contextual considerations into teaching, ensuring relevance to local societal norms.
4. Actively involve students as coproducers of curricula, aligning learning experiences with their lived realities.

By centering student perspectives and implementing these strategies, professionalism curricula can more effectively prepare future physicians for context-aware, patient-centered practice, ultimately enhancing patient experience and care quality.

## Highlights

- Student perspectives drive curriculum relevance**:** Undergraduate learners offer critical insights into how professionalism is experienced and learned, informing more effective teaching strategies.
- Role modeling is essential: Senior clinicians’ behaviors strongly shape learners’ professional identity and should be integrated through structured observation and feedback.
- Reflective practice and patient narratives enhance learning: Narrative exercises and reflection link professional values to real patient experiences, supporting empathy, communication, and ethical decision-making.
- Cultural and contextual awareness matters: Professionalism curricula should consider local societal norms and institutional culture to ensure learning is meaningful and applicable.
- Actionable strategies improve patient-centered care: Embedding students’ voices, structured supervision, reflection, and culturally responsive practices in curricula can strengthen professionalism and positively impact patient experience.

## Supplemental Material

sj-docx-1-jpx-10.1177_23743735251401814 - Supplemental material for Student Perspectives on Professionalism: Time to Reform Curriculum for Better Patient Experience Supplemental material, sj-docx-1-jpx-10.1177_23743735251401814 for Student Perspectives on Professionalism: Time to Reform Curriculum for Better Patient Experience by Nagina Khan, Sucheta Tiwari, Walther van Mook, Subodh Dave, Sohyun Ha, Joshua Sagisi, Marie Hickman, Nicole Davi, Chantel Aftab, Salim Al-Huseini and Wolfgang Gilliar in Journal of Patient Experience

sj-docx-2-jpx-10.1177_23743735251401814 - Supplemental material for Student Perspectives on Professionalism: Time to Reform Curriculum for Better Patient Experience Supplemental material, sj-docx-2-jpx-10.1177_23743735251401814 for Student Perspectives on Professionalism: Time to Reform Curriculum for Better Patient Experience by Nagina Khan, Sucheta Tiwari, Walther van Mook, Subodh Dave, Sohyun Ha, Joshua Sagisi, Marie Hickman, Nicole Davi, Chantel Aftab, Salim Al-Huseini and Wolfgang Gilliar in Journal of Patient Experience

## Abbreviations

ERIC: education resources information center
MMAT: mixed methods appraisal tool
PRISMA: preferred reporting items for systematic reviews and meta-analyses
UME: undergraduate medical education
