# Supplementary material for: Student Perspectives on Professionalism: Time to Reform Curriculum for Better Patient Experience
Source: J Patient Exp. 2025 Dec 22;12:23743735251401814. doi: 10.1177/23743735251401814 (PMC12722648; doi:10.1177/23743735251401814)
Supplement: sj-docx-1-jpx-10.1177_23743735251401814 - Supplemental material for Student Perspectives on Professionalism: Time to Reform Curriculum for Better Patient Experience [file sj-docx-1-jpx-10.1177_23743735251401814.docx]

**Table 3.** Good Practices in Professionalism Education: Evidence, Curricular Applications and Measurable Outcomes.

| **Practice Identified by Students** | **Description / Evidence from Studies** | **Suggested Curricular Application** | **Potential Measurable Outcomes** |
| --- | --- | --- | --- |
| **Narrative medicine and reflection** | Reflective journaling, essays, and storytelling enhanced empathy, ethics, and identity formation (Arntfield et al., 2013; Dhaliwal et al., 2018). | Embed structured reflective writing sessions into clinical rotations; use guided prompts. | Increased empathy scores: improved reflective capacity (measured via validated scales). |
| **Role modeling and supervision** | Senior clinicians’ behavior strongly influenced student professionalism; both positive and negative models noted.^18,27^ | Faculty development for explicit role modeling; structured observation with feedback. | Student-reported quality of role modeling; alignment between observed and taught values. |
| **Patient narratives & early contact** | Direct engagement with patients normalized a patient-centered identity and fostered empathy.^28^ | Incorporate expert patient teachers and early patient contact into preclinical years. | Patient satisfaction with student interactions; student empathy ratings. |
| **Small-group discussion of critical incidents** | Peer and faculty-facilitated discussions encouraged reflection on professionalism challenges.^20,25^ | Regularly scheduled professionalism ‘case rounds’ or ethics discussions. | Depth of reflection (Rubric-based); confidence in handling ethical dilemmas. |
| **Teamwork and collaboration exercises** | Students linked professionalism to communication and accountability in teams.^29^ | Interprofessional simulation sessions with structured debriefs. | Teamwork assessment scores; peer evaluations. |
| **Cultural responsiveness** | Professional identity shaped by local culture and societal expectations.^22^ | Integrate modules on sociocultural influences in professionalism. | Student cultural humility/self-awareness scores. |
