# Supplementary material for: Student Perspectives on Professionalism: Time to Reform Curriculum for Better Patient Experience
Source: J Patient Exp. 2025 Dec 22;12:23743735251401814. doi: 10.1177/23743735251401814 (PMC12722648; doi:10.1177/23743735251401814)
Supplement: sj-docx-2-jpx-10.1177_23743735251401814 - Supplemental material for Student Perspectives on Professionalism: Time to Reform Curriculum for Better Patient Experience [file sj-docx-2-jpx-10.1177_23743735251401814.docx]

**Supplementary Material**

**Table. 1: Search strategy**

**Literature search**

The populations of interest are undergraduate medical students and professionals involved in teaching and assessing professionalism in undergraduate medical education. These include medical educators and leaders, professors, lecturers, clinicians, mentors, advisors, and/or supervisors in academic institutions, community practice settings, hospitals, and medical schools.

PubMed (Medline), Embase, Scopus, PsycINFO, and ERIC will be searched for studies published in English between 2010 and 2020. Searches were rerun for 2021-2023.

**Medline Search Strategy**

|  | **Search terms** | **Number of references in original search** | **Number of references 2021-2023** |
| --- | --- | --- | --- |
| 1 | exp PROFESSIONALISM/ | 1419 | 444 |
| 2 | "ETHICS, PROFESSIONAL"/ | 6959 | 59 |
| 3 | "EDUCATION, MEDICAL, UNDERGRADUATE"/ | 25253 | 2257 |
| 4 | "EDUCATION, MEDICAL"/ | 58435 | 3250 |
| 5 | exp "STUDENTS, MEDICAL"/ | 37040 | 7858 |
| 6 | ("medical education" OR "medical teach*").ti,ab | 44109 | 9845 |
| 7 | (student* OR undergrad*).ti,ab | 316378 | 85972 |
| 8 | ("medical student*").ti,ab | 43963 | 11961 |
| 18 | (professionalism).ti,ab | 7639 | 1652 |
| 19 | (1 OR 2 OR 18) | 14992 | 1763 |
| 20 | (4 OR 6) | 89186 | 10772 |
| 21 | (5 OR 7 OR 8) | 324273 | 86399 |
| 22 | (20 AND 21) | 23575 | 4654 |
| 23 | (3 OR 22) | 44311 | 6133 |
| 24 | (19 AND 23) | 1032 | 160 |
| 25 | 24 [DT 2010-2021] [Languages English] | 668 | 157 |

**Embase Search Strategy**

|  | **Search terms** | **Number of references in original search** | **Number of references 2021-2023** |
| --- | --- | --- | --- |
| 26 | PROFESSIONALISM/ | 9752 | 2056 |
| 27 | (professionalism).ti,ab | 9314 | 1787 |
| 28 | (26 OR 27) | 14676 | 2625 |
| 29 | exp "MEDICAL EDUCATION"/ | 330962 | 50278 |
| 30 | exp "MEDICAL STUDENT"/ | 74913 | 18791 |
| 31 | ("medical education" OR "medical teach*").ti,ab | 50423 | 11654 |
| 32 | (student* OR undergrad*).ti,ab | 413063 | 96469 |
| 33 | ("medical student*").ti,ab | 57918 | 15719 |
| 34 | (29 OR 31) | 340742 | 52140 |
| 35 | (30 OR 32 OR 33) | 433111 | 100328 |
| 36 | (34 AND 35) | 80285 | 15462 |
| 37 | (28 AND 36) | 2161 | 377 |
| 38 | 37 [DT 2010-2021] [English language] | 1516 | 369 |

**PsycInfo Search Strategy**

|  | **Search terms** | **Number of references in original search** | **Number of references 2021-2023** |
| --- | --- | --- | --- |
| 39 | PROFESSIONALISM/ | 3958 | 346 |
| 40 | (professionalism).ti,ab | 4473 | 494 |
| 41 | (39 OR 40) | 6620 | 664 |
| 42 | exp "MEDICAL EDUCATION"/ | 24925 | 1968 |
| 43 | "MEDICAL STUDENTS"/ | 13795 | 1196 |
| 44 | ("medical education" OR "medical teach*").ti,ab | 7635 | 679 |
| 45 | (student* OR undergrad*).ti,ab | 591980 | 63737 |
| 46 | ("medical student*").ti,ab | 13649 | 1242 |
| 47 | (42 OR 44) | 26934 | 2162 |
| 48 | (43 OR 45 OR 46) | 593557 | 63801 |
| 49 | (47 AND 48) | 13109 | 1026 |
| 50 | (41 AND 49) | 524 | 22 |
| 51 | 50 [DT 2010-2021] [Languages English] | 353 | 21 |

| **Number of references in original search** | **Number of references 2022-2023** |
| --- | --- |
| **ERIC Search strategy** | **ERIC Search strategy** |
| professionalism and medical and (student or undergraduate), limit to last 10 years | professionalism and medical and (student or undergraduate), limit 2022-2024 |
| 76 references retrieved | 35 references retrieved |
|  |  |
| **Total references retrieved = 2,613** | **Total references retrieved = 582** |
| **Removed duplicates = 1,858 references** | **Removed duplicates (including 2021 articles in original results) = 382 references**  **Removed 74 review articles and anything where teaching or assessing professionalism in medical students was not mentioned.**    **Total: There were 113 references** |

**Table. 2: The articles included in the review on professionalism and learners’ experiences**

|  | Abdalla M, Dash N, Shorbagi S, Ahmed M. Preclerkship medical students’ perceptions of medical professionalism in the College of Medicine-University of Sharjah: A vision to sustain success in medical education. J Educ Health Promot. 2020;9(1). |
| --- | --- |
|  | Abrams MP, Eckert T, Topping D, Daly KD. Reflective Writing on the Cadaveric Dissection Experience: An Effective Tool to Assess the Impact of Dissection on Learning of Anatomy, Humanism, Empathy, Well-Being, and Professional Identity Formation in Medical Students. Anat Sci Educ. 2021;14(5):658–65. |
|  | Adema M, Dolmans DHJM, Raat JAN, Scheele F, Jaarsma ADC, Helmich E. Social Interactions of Clerks: The Role of Engagement, Imagination, and Alignment as Sources for Professional Identity Formation. Acad Med. 2019;94(10):1567–73. |
|  | Al-Abdulrazzaq D, Al-Fadhli A, Arshad A. Advanced medical students’ experiences and views on professionalism at Kuwait University. BMC Med Educ [Internet]. 2014;14:150. |
|  | Arntfield SL, Slesar K, Dickson J, Charon R. Narrative medicine as a means of training medical students toward residency competencies. Patient Educ Couns. 2013;91(3):280–6. |
|  | Azmand S, Ebrahimi S, Iman M, Asemani O. Learning professionalism through hidden curriculum: Iranian medical students’ perspective. J Med Ethics Hist Med. 2018;11. |
|  | Barr J, Bull R, Rooney K. Developing a patient focussed professional identity: An exploratory investigation of medical students’ encounters with patient partnership in learning. Adv Heal Sci Educ. 2014;20(2):325–38. |
|  | Bennett D, Mccarthy M, O’Flynn S, Kelly M. In the eye of the beholder: Student perspectives on professional roles in practice. Med Educ. 2013;47(4):397–407. |
|  | Borgstrom E, Cohn S, Barclay S. Medical professionalism: Conflicting values for tomorrow’s doctors.  J Gen Intern Med. 2010;25(12):1330–6. |
|  | Braun UK, Gill AC, Teal CR, Morrison LJ. The utility of reflective writing after a palliative care experience: Can we assess medical students’ professionalism? J Palliat Med [Internet]. 2013;16(11):1342–9. |
|  | Butani L, Bogetz A, Plant J. Illuminating exemplary professionalism using appreciative inquiry dialogues between students and mentors; 29801424. Med Teach [Internet]. 2019;41(3):325–31. |
|  | Cusimano MC, Ting DK, Kwong JL, Van Melle E, MacDonald SE, Cline C. Medical Students Learn Professionalism in Near-Peer Led, Discussion-Based Small Groups; 30554529. Teach Learn Med [Internet]. 2019;31(3):307–18. |
|  | Curry SE, Cortland CI, Graham MJ, Cortl, Graham MJ. Role-modelling in the operating room: Medical student observations of exemplary behaviour. Med Educ [Internet]. 2011;45(9):946–57. |
|  | Dhaliwal U, Singh S, Singh N. Reflective student narratives: honing professionalism and empathy. Indian J Med Ethics [Internet]. 2018;3(1):9–15. |
|  | Finn G, Garner J, Sawdon M. “You’re judged all the time” Students’ views on professionalism: A multicentre study. Med Educ [Internet]. 2010;44(8):814–25. |
|  | Fredholm A, Manninen K, Hjelmqvist H, Silén C. Authenticity made visible in medical students’ experiences of feeling like a doctor. Int J Med Educ. 2019;10:113–21. |
|  | Ginsburg S, Lingard L. “Is that normal?” Pre‐clerkship students’ approaches to professional dilemmas. Med Educ [Internet]. 2011;45(4):362–71. |
|  | Gonsalves C, Zaidi Z. Hands in medicine: understanding the impact of competency-based education on the formation of medical students’ identities in the United States. J Educ Eval Heal Prof [Internet]. 2016;13:31. |
|  | Haffling A-C, Beckman A, Pahlmblad A, Edgren G. Students’ reflections in a portfolio pilot: Highlighting professional issues. Med Teach [Internet]. 2010;32(12):e532–40. |
|  | Hultman CS, Connolly A, Halvorson EG, Rowland P, Meyers MO, Mayer DC, et al. Get on your boots: Preparing fourth-year medical students for a career in surgery, using a focused curriculum to teach the competency of professionalism; 22878148. J Surg Res [Internet]. 2019;177(2):217–23. |
|  | Karnieli-Miller O, Taylor AC, Cottingham AH, Inui TS, Vu TR, Frankel RM. Exploring the meaning of respect in medical student education: An analysis of student Narratives. J Gen Intern Med. 2010;25(12):1309–14. |
|  | Karnieli-Miller O, Vu TR, Frankel RM, Holtman MC, Clyman SG, Hui SL, et al. Which experiences in the hidden curriculum teach students about professionalism? Acad Med [Internet]. 2011;86(3):369–77. |
|  | Kaul B, Teal CR, Greenberg SB. Lapses in medical professionalism: A lack of consensus on appropriate sanctions. J Gen Intern Med [Internet]. 2014;29:S136–7. |
|  | Kavas MV, Demirören M, Koşan AMA, Karahan ST, Yalim NY. Turkish students’ perceptions of professionalism at the beginning and at the end of medical education: A cross-sectional qualitative study. Med Educ Online. 2015;20(1). |
|  | Kittmer T, Hoogenes J, Pemberton J, Cameron BH. Exploring the hidden curriculum: A qualitative analysis of clerks’ reflections on professionalism in surgical clerkship. Am J Surg [Internet]. 2013;205(4):426–33. |
|  | Kong WM, Knight S. Bridging the education-action gap: A near-peer case-based undergraduate ethics teaching programme. J Med Ethics. 2017;43(10):692–6. |
|  | Langendyk V, Mason G, Wang S. How do medical educators design a curriculum that facilitates student learning about professionalism? Int J Med Educ [Internet]. 2016;7:32–43. |
|  | Lutz Gabriele, Scheffer Christian, Edelhaeuser Friedrich, Tauschel Diethard NM. A reﬂective practice intervention for professional development, reduced stress and improved patient care—A qualitative developmental evaluation. Patient Educ Couns. 2013;92:337–45. |
|  | Maitra A, Lin S, Rydel TA, Schillinger E. Balancing forces: Medical students’ reflections on professionalism challenges and professional identity formation. Fam Med. 2021;53(3):200–6. |
|  | Mak-van der Vossen M, Teherani A, van Mook WNKA, Croiset G, Kusurkar RA. Investigating US medical students’ motivation to respond to lapses in professionalism. Med Educ. 2018;52(8):838–50. |
|  | McEvoy M, Butler B, MacCarrick G. {Teaching professionalism through virtual means.}. Clin Teach. 2012;9(1):32–6. |
|  | Mirmoghtadaie Z, Ahmady S, Kohan N, Rakhshani T. An interesting result of a qualitative research: Academic exhaustion barrier to professionalism in medical students. J Educ Health Promot. 2020;9(1). |
|  | Monrouxe L V., Rees CE, Hu W. Differences in medical students’ explicit discourses of professionalism: Acting, representing, becoming. Med Educ. 2011;45(6):585–602. |
|  | Pierce JR, Lopez N, Sbar E. Teaching professionalism in the clinical setting: Testing a practical structured model. South Med J. 2020;113(12):640–2. |
|  | Prunuske A, Houss BA, Kosobuski AW. Alignment of roles of near-peer mentors for medical students underrepresented in medicine with medical education competencies: A qualitative study. BMC Med Educ. 2019;19(1). |
|  | Ramakrishna J, Valani R, Sriharan A, Scolnik D. Design and pilot implementation of an evaluation tool  assessing professionalism, communication and collaboration during a unique global health elective. Med Confl Surviv. 2014;30(1):56–65. |
|  | Ribeiro DL, Costa M, Helmich E, Jaarsma D, de Carvalho-Filho MA. ‘I found myself a despicable being!’: Medical students face disturbing moral dilemmas. Med Educ. 2021;55(7):857–71. |
|  | Safari Y, Khatony A, Khodamoradi E, Rezaei M. The role of hidden curriculum in the formation of professional ethics in Iranian medical students: A qualitative study. J Educ Health Promot. 2020;9(1). |
|  | Shamim MS, Zubairi NA, Sayed MH, Gazzaz ZJ. Innovation in ethics and professionalism course: Early experience with portfolio-workbook. J Pak Med Assoc [Internet]. 2016;66(9):1149–53. |
|  | Shaw MK, Rees CE, Andersen NB, Black LF, Monrouxe L V. Professionalism lapses and hierarchies: A qualitative analysis of medical students’ narrated acts of resistance. Soc Sci Med. 2018;219:45–53. |
|  | Shevell AH, Thomas A, Fuks A. Teaching professionalism to first year medical students using video clips. Med Teach. 2015;37(10):935–42. |
|  | Shield RR, Farrell TW, Campbell SE, Nanda A, Wetle T. Professional Development and Exposure to Geriatrics: Medical Student Perspectives From Narrative Journals. Gerontol Geriatr Educ. 2015;36(2):144–60. |
|  | Shield RR, Tong I, Tomas M, Besdine RW. Teaching communication and compassionate care skills: An innovative curriculum for pre-clerkship medical students. Med Teach. 2011;33(8):416. |
|  | Shiozawa T, Glauben M, Banzhaf M, Griewatz J, Hirt B, Zipfel S, et al. An Insight into Professional Identity Formation: Qualitative Analyses of Two Reflection Interventions During the Dissection Course. Anat Sci Educ. 2020;13(3):320–32. |
|  | Sitnik, E., Bertoch, B., Roseamelia, C. & Germain, L. J. An Introductory Qualitative Exploration of Medical Student Perceptions of Professionalism at One Medical University. *PRiMER* 2023; **7**. |
|  | Song, X. & Elftman, M. Professionalism in small group learning between face-to-face and virtual settings: a mixed-methods study. Int J Med Educ 2023; 14, 36–42. |
|  | Stockley AJ, Forbes K. Medical professionalism in the formal curriculum: 5th year medical students’ experiences. BMC Med Educ [Internet]. 2014;14(1):259. |
|  | Stubbing EA, Helmich E, Cleland J. Medical student views of and responses to expectations of professionalism. Med Educ. 2019;53(10):1025–36. |
|  | Tucker CR, Choby BA, Moore A, Parker RS, Zambetti BR, Naids S, et al. Speaking up: Using OSTEs to understand how medical students address professionalism lapses. Med Educ Online. 2016;21(1). |
|  | Varga-Atkins T, Dangerfield P, Brigden D. Developing professionalism through the use of wikis: A study with first-year undergraduate medical students. Med Teach. 2010;32(10):824–9. |
|  | Wang XM, Swinton M, You JJ. Medical students’ experiences with goals of care discussions and their impact on professional identity formation. Med Educ. 2019;53(12):1230–42. |
|  | Whelan B, Hjörleifsson S, Schei E. Shame in medical clerkship: “You just feel like dirt under someone’s shoe.” Perspect Med Educ. 2021;10(5):265–71. |
|  | Yoon MH, Blatt BC, Greenberg LW. Medical Students’ Professional Development as Educators Revealed Through Reflections on Their Teaching Following a Students-as-Teachers Course. Teach Learn Med. 2017;29(4):411–9. |
|  | Youssef VC, Peters D, Youssef FF. “It’s All About How You Carry Yourself About”: How Medical Students Conceptualize Professionalism in Trinidad & Tobago. Teach Learn Med. 2016;28(4):367–74. |

**Table 3: Calculation of per cent agreement on the critical appraisal rating**

**Var# Raters Difference**

**ST NK**

1. 1 1 1
2. 1 1 1
3. 1 1 1
4. 0 1  -1
5. 1 1 1
6. 1 1 1
7. 1 1 1
8. 1 1 1
9. 0 1 -1
10. 1 1 1
11. 1 1 1
12. 1 1 1
13. 1 1 1
14. 1 1 1
15. 1 1 1
16. 1 1 1
17. 1 1 1
18. 1 1 1
19. 1 1 1
20. 1 1 1
21. 1 1 1
22. 1 1 1
23. 1 1 1
24. 1 1 1
25. 1 1 1
26. 1 1 1
27. 1 1 1
28. 1 1 1
29. 1 1 1
30. 1 1 1
31. 1 1 1
32. 1 1 1
33. 1 1 1
34. 1 1 1
35. 1 1 1
36. 1 1 1
37. 1 1 1
38. 1 1 1
39. 1 1 1
40. 1 1 1
41. 1 1 1
42. 1 1 1
43. 1 1 1
44. 1 1 1
45. 1 1 1
46. 1 1 1
47. 1 1 1
48. 1 1 1
49. 1 1 1
50. 1 1 1
51. 1 1 1
52. 1 1 1
53. 1 1 1
54. 1 1 1

**Percent agreement for two raters**

Number of ratings in agreement 52

Total number of ratings 54

Percent Agreement **96%**
